# Bioengineered Hydrogels Recapitulate Fibroblast Heterogeneity in Cancer

**DOI:** 10.1002/advs.202307129

**Published:** 2024-03-17

**Authors:** Nicholas Ching Wei Ho, Josephine Yu Yan Yap, Zixuan Zhao, Yunyun Wang, Kanishka Fernando, Constance H Li, Xue Lin Kwang, Hong Sheng Quah, Camille Arcinas, N. Gopalakrishna Iyer, Eliza Li Shan Fong

**Affiliations:** ^1^ Translational Tumor Engineering Laboratory, Department of Biomedical Engineering National University of Singapore Singapore 119276 Singapore; ^2^ The N.1 Institute for Health National University of Singapore Singapore 117456 Singapore; ^3^ Cancer Therapeutics Research Laboratory National Cancer Centre Singapore Singapore 168583 Singapore; ^4^ Duke‐NUS Medical School National University of Singapore Singapore 169857 Singapore; ^5^ Cancer Science Institute National University of Singapore Singapore 117599 Singapore

**Keywords:** CAF heterogeneity, cancer‐associated fibroblasts, hydrogel, inflammatory CAF, myofibroblastic CAF

## Abstract

Recently mapped transcriptomic landscapes reveal the extent of heterogeneity in cancer‐associated fibroblasts (CAFs) beyond previously established single‐gene markers. Functional analyses of individual CAF subsets within the tumor microenvironment are critical to develop more accurate CAF‐targeting therapeutic strategies. However, there is a lack of robust preclinical models that reflect this heterogeneity in vitro. In this study, single‐cell RNA sequencing datasets acquired from head and neck squamous cell carcinoma tissues to predict microenvironmental and cellular features governing individual CAF subsets are leveraged. Some of these features are then incorporated into a tunable hyaluronan‐based hydrogel system to culture patient‐derived CAFs. Control over hydrogel degradability and integrin adhesiveness enabled derivation of the predominant myofibroblastic and inflammatory CAF subsets, as shown through changes in cell morphology and transcriptomic profiles. Last, using these hydrogel‐cultured CAFs, microtubule dynamics are identified, but not actomyosin contractility, as a key mediator of CAF plasticity. The recapitulation of CAF heterogeneity in vitro using defined hydrogels presents unique opportunities for advancing the understanding of CAF biology and evaluation of CAF‐targeting therapeutics.

## Graphical abstract

In this study, Ho and co‐workers leveraged the use of bioengineered hyaluronan hydrogels to recapitulate cancer‐associated fibroblast (CAF) heterogeneity in vitro in head and neck squamous cell carcinoma. Tuning hydrogel degradation alters microtubule dynamics in CAFs, which plays a key role in the plasticity between myofibroblastic and inflammatory CAF subsets.

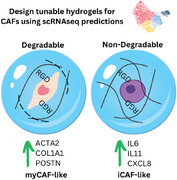

## 1 Introduction

In recent years, there is increasing evidence that high cancer‐associated fibroblast (CAF) content in patient tumors is associated with poor prognosis in different cancer types.^[^ ^1^ ^]^ It is well‐established that CAFs perform numerous pro‐tumorigenic functions in the tumor microenvironment (TME) that facilitate tumor progression and metastasis, such as promotion of cancer cell growth, angiogenesis, and extracellular matrix (ECM) remodeling.^[^ ^2^ ^]^ Additionally, CAFs also orchestrate an immunosuppressive TME and are associated with resistance to immunotherapy.^[^ ^3^, ^4^ ^]^ Given their abundance in tumors and significance in drug resistance, efforts have been made to target CAFs. Yet, several studies have reported how depletion of CAFs in genetically engineered mouse models of pancreatic cancer unexpectedly resulted in poorly differentiated and aggressive tumors, and consequently, worse survival.^[^ ^5^, ^6^ ^]^ Clinical trials targeting CAFs through inhibition of the Hedgehog pathway in pancreatic cancer also showed dismal results.^[^ ^7^, ^8^, ^9^ ^]^ In contrast, targeting stromal Hedgehog signaling has proven to be beneficial in other cancer types.^[^ ^10^ ^]^ These contradictory data across different studies may likely be a consequence of the inherent diversity of CAFs.

The advent of single‐cell RNA sequencing (scRNAseq) has revolutionized our understanding of the extent of CAF heterogeneity beyond single gene markers, with recent studies revealing the transcriptomic diversity of CAFs.^[^ ^2^ ^]^ Underlying these is a common observation that at least three distinct CAF subsets (or states) exist, myofibroblastic CAFs (myCAFs), inflammatory CAFs (iCAFs), and antigen‐presenting CAFs (apCAFs).^[^ ^2^ ^]^ While iCAFs are associated with cytokine and chemokine secretion, and immunomodulation, myCAFs are associated with extracellular matrix (ECM) modulation, collagen deposition, contraction, and adhesion.^[^ ^2^ ^]^ apCAFs are a small population of CAFs involved in antigen presentation, and could be associated with improved immunotherapy outcomes.^[^ ^11^ ^]^ As previous studies have already suggested how non‐specific targeting or deletion of fibroblasts may lead to detrimental outcomes,^[^ ^12^ ^]^ intense efforts are now underway to delineate the functions of each CAF subset to develop more accurate CAF‐targeting strategies to achieve clinical benefit in specific clinical and therapeutic contexts. Unfortunately, methods to robustly culture myCAFs and iCAFs in vitro for such functional studies remain limited.^[^ ^13^, ^14^, ^15^ ^]^

Previous studies on fibroblasts suggest these cells are highly sensitive to in vitro culture conditions. Fibroblasts in three‐dimensional collagen matrices adopt different morphologies, from dendritic to bipolar, depending on the level of matrix stiffness and tension.^[^ ^16^ ^]^ The presence of cell‐cell adhesion also appears to be important, as floating fibroblast spheroids are senescent, secrete various cytokines and chemokines, and alter cancer cell invasiveness.^[^ ^17^ ^]^ More recently, various groups reported how hydrogel stiffness and degradability can influence YAP/TAZ signaling in both encapsulated mesenchymal stem cells^[^ ^18^ ^]^ and fibroblasts.^[^ ^19^ ^]^ Taken together, these findings suggest the possibility of deriving different CAF subsets in vitro by modulating matrix properties.

In this study, to generate myCAFs and iCAFs in vitro, we leveraged transcriptomic datasets acquired from patient‐derived head and neck squamous cell carcinoma (HNSCC) tumors to guide hydrogel design. We show that hydrogel degradability and integrin adhesiveness are critical matrix parameters that robustly support the derivation of different CAF subsets.

## 2 Results

### 2.1 scRNAseq Analysis Reveals the Presence of 4 CAF Subsets in HNSCC

**Figure 1:**
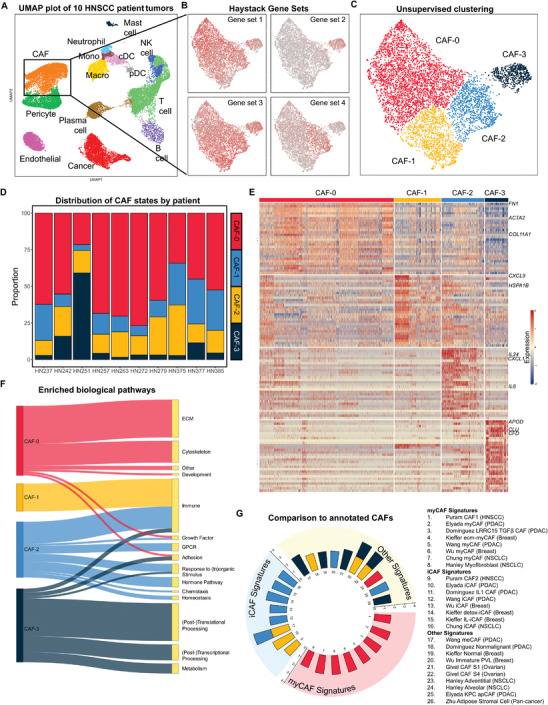
Single‐cell RNA sequencing of 10 HNSCC patient tumors reveals 4 transcriptionally‐distinct CAF subsets. A) UMAP plot of cells of primary site tumors from 10 HNSCC patients. B) CAFs were identified from A) and re‐plotted on a CAF‐specific UMAP. Following which, single CellHaystack was used to identify gene sets that varied across the 2D UMAP space, of which 4 were settled upon. C) Unsupervised clustering based on the CAF‐specific UMAP plot to the resolution of 4 clusters (CAF‐0 to −3), which was found to be similar to the distribution of the 4 gene sets in B). D) Distribution of the 4 CAF subsets across patients. E) Heatmap of differentially expressed genes across the 4 CAF subsets. F) Sankey diagram whose link widths represent the number of biological pathways enriched by each CAF subset (left nodes), for each category of pathways (right nodes). Enrichment was computed using GSEA. G) Normalized enrichment scores (calculated using GSEA) of each CAF subset for gene signatures collected from past studies. More information regarding each study can be found under Experimental Section. PDAC – pancreatic ductal adenocarcinoma; NSCLC – non‐small cell lung cancer.

We identified fibroblasts from a scRNAseq dataset acquired from 10 HNSCC patient tumors using fibroblast‐associated markers such as *COL1A1* and *ACTA2* (**Figure** **1**A; Table S1, Supporting Information). We then applied singleCellHaystack^[^ ^20^ ^]^ to identify gene sets that varied in expression across the 2D UMAP space of 6044 CAFs, and derived unique gene sets that exist within the fibroblast population (Figure 1B). Unsupervised Louvain clustering algorithm revealed 4 subclusters (CAF‐0 to −3), whose positions matched the regions differing in gene set expression that were independently determined by singleCellHaystack (Figure 1B,C). Importantly, these clusters were found in all patients (Figure 1D).

Analyses of the differentially expressed genes (DEGs; Figure 1E; Table S2, Supporting Information) showed that the CAF‐0 subset appeared to have higher expression of myCAF‐associated genes: *FN1*, *ACTA2*, and *COL1A1*. Conversely, the CAF‐1 (high *CXCL9*) and CAF‐2 (high *IL6*, *CXCL1*, and *IL24*) subsets upregulated various cytokines and chemokines characteristic of the iCAF phenotype. The smallest subcluster, CAF‐3, expressed genes associated with the complement pathway (*CFD*) and lipid metabolism (*APOD*). Gene set enrichment analysis (GSEA) was performed using gene sets from GO:BP (Gene Ontology: Biological Processes),^[^ ^21^ ^]^ KEGG (Kyoto Encyclopedia of Genes and Genomes),^[^ ^22^ ^]^ and Reactome.^[^ ^23^ ^]^ CAF‐0 was characterized by positive enrichment of many ECM and cytoskeletal pathways, commonly found in myCAFs (Figure 1F; Table S3, Supporting Information). In contrast, CAF‐1 to −3 showed positive enrichment of immune‐related pathways: CAF‐1 and CAF‐2 were more related to IFN‐ and IL‐signalling, respectively, while the complement pathway was activated in CAF‐3. In addition, CAF‐3 was associated with pathways in metabolism and RNA and protein processing.

To validate the 4 CAF subsets identified in HNSCC here, we compared these with gene signatures of CAF subsets from 10 other studies (Experimental Section) across 5 different cancer types (Figure 1G).^[^ ^14^, ^24^, ^25^, ^26^, ^27^, ^28^, ^29^, ^30^, ^31^, ^32^ ^]^ Using GSEA, CAF‐0 was the only subset associated with enrichment of myCAF gene signatures, while CAF‐1 and −2 both showed positive enrichment for iCAF signatures. CAF‐3 upregulated *CD74* but not the apCAF gene signature. Instead, CAF‐3 appeared most similar to adipose stromal cells (ASCs) previously described as progenitors of myCAFs.^[^ ^32^ ^]^ CAF‐3 also upregulated *DPT* (Table S2, Supporting Information), a marker of CAF progenitors discovered in a pan‐tissue fibroblast analysis.^[^ ^33^ ^]^ Hence, based on these analyses, we will refer to CAF‐0 to −3 as myCAFs, IFN‐iCAFs, IL‐iCAFs, and adipose‐like CAFs (adipoCAFs), respectively.

### 2.2 Microenvironmental and Cellular Features of CAF Subsets may be Predicted Using scRNAseq Data

**Figure 2:**
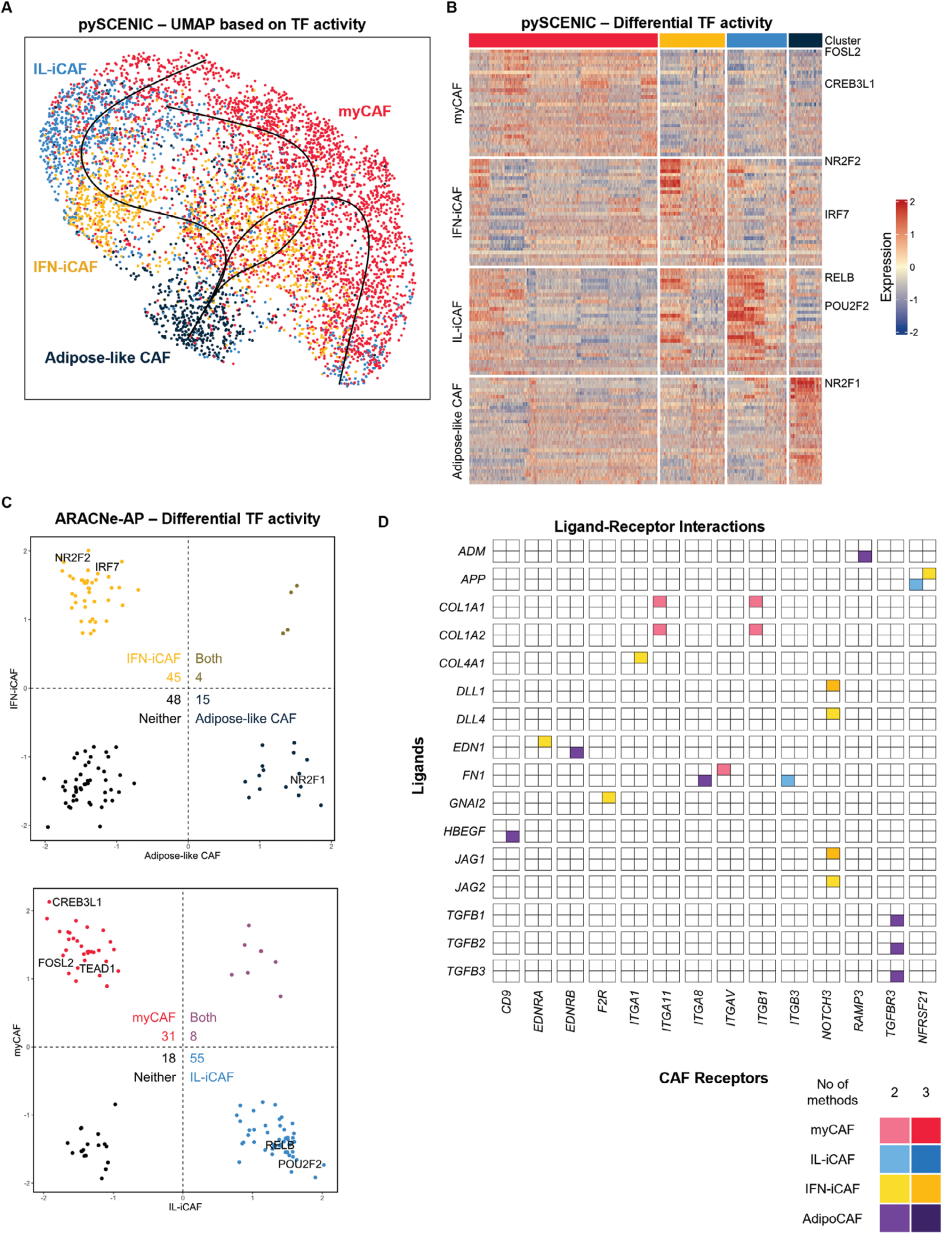
CAF subsets are distinct in transcription factor activities and ligand‐receptor interactions. A) UMAP plot of CAFs based on pySCENIC‐predicted transcription factor activities. Lines represent pseudotime analysis using adipoCAFs as the progenitor cluster, showing various possible routes of polarization. B) Heatmap of differentially active transcription factors across the 4 CAF subsets, as predicted by pySCENIC. C) Scatter plots depict how CAF subsets compared to each other based on ARACNe‐AP‐predicted transcription factor activities. The positive region of each axis indicates transcription factors that are predicted to regulate positive markers of the respective CAF subset, and vice versa for the negative region. Top plot compares adipoCAFs and IFN‐iCAFs, while bottom plot compares myCAFs and IL‐iCAFs. D) Plot reflects the top ligand‐receptor interactions for the 4 CAF subsets, where the shade of the square corresponds to the number of methods (out of 3) that predicted the same interaction.

Having identified the different CAF subsets in HNSCC, we hypothesized that predicting transcription factor (TF) activities and ligand‐receptor interactions may reveal microenvironmental cues that contribute to the identity of each CAF subset. It is now well‐established that physical and chemical stimuli in the cell microenvironment can influence TF activity.^[^ ^34^, ^35^ ^]^ We therefore used pySCENIC^[^ ^36^ ^]^ and ARACNe‐AP^[^ ^37^ ^]^ to predict TF activities in the different CAF subsets. Re‐plotting the CAF data based on pySCENIC‐predicted TF activities revealed that the 4 CAF clusters were largely preserved (**Figure** **2**A). We also performed trajectory analysis using the Slingshot algorithm^[^ ^38^ ^]^ on the pySCENIC‐derived UMAP, where the adipoCAF subset served as the starting cluster (as a putative progenitor population^[^ ^32^ ^]^). We performed this trajectory analysis to determine whether myCAFs and IL‐iCAFs could potentially transition into each other, as was previously reported.^[^ ^13^ ^]^ CAF plasticity would imply that cell‐extrinsic microenvironmental cues can influence CAFs to switch between different phenotypic states. Of the three predicted trajectories, two ended in myCAFs and one in IL‐iCAF, with all passing through IFN‐iCAFs. This suggests that IFN‐iCAFs are an intermediate subset, which potentially gives rise to both myCAFs and IL‐iCAFs (Figure 2A). Importantly, the TF trajectories also suggest plasticity between the myCAF and IL‐iCAF states, highlighting the possibility of leveraging CAF subset‐specific exogenous cues to manipulate myCAFs into IL‐iCAFs and vice versa.

Differential TF activity analysis (Figure 2B; Table S4, Supporting Information) by pySCENIC showed that myCAFs had higher *FOSL2* and *CREB3L1* activities while IL‐iCAFs showed higher *RELB* and *POU2F2* activities. *CREB3L1* is known to upregulate ECM‐ and myCAF‐associated genes such as *COL1A1*,^[^ ^39^ ^]^ while active JAK‐STAT signaling and NF‐κB activity (via *RELB*) have been reported for iCAFs.^[^ ^40^ ^]^ For the other two clusters, we observed a switch from *NR2F1* to *NR2F2* activity as adipoCAFs transit to IFN‐iCAFs. These findings were confirmed using ARACNe‐AP, by conducting GSEA on CAF cluster markers and using the predicted target genes of each TF as gene sets. By investigating the normalized enrichment scores (NES) of each TF's targets and doing a pairwise comparison of CAF clusters that can transition from one to another (Figure 2C; Table S4, Supporting Information), the same TFs were observed to associate with upregulating markers of each CAF subset. Importantly, ARACNe‐AP also implicated the role of *TEAD1* in myCAFs (Figure 2C). Together with YAP via the Hippo pathway, TEAD family proteins are commonly associated with high cellular tension brought about by actomyosin contractility, a hallmark of myofibroblasts.^[^ ^41^, ^42^ ^]^ Further analysis on the ligand‐receptor interactions associated with each CAF subset using CellPhoneDB,^[^ ^44^ ^]^ NATMI,^[^ ^45^ ^]^ and Scriabin,^[^ ^46^ ^]^ revealed that all CAF subsets are involved in ECM‐integrin interactions. However, these interactions are more prominent in myCAFs compared to other CAF subsets (Figure 2D; Table S5, Supporting Information).

Together, these analyses suggest the need to provide conditions promoting cellular tension and integrin adhesion for the derivation of myCAFs. Accordingly, we hypothesized that a degradable hydrogel with integrin‐binding motifs would promote cell spreading and generate cellular contractility in encapsulated CAFs, thereby supporting the myCAF phenotype. Conversely, the iCAF phenotype could either be induced in a hydrogel without integrin‐binding motifs to prevent generation of cellular tension, or in a non‐degradable hydrogel to prevent cell spreading.

### 2.3 Different Hydrogel Conditions Induce Unique Changes in CAF Morphology

Using a thiolated HA hydrogel system conducive for cancer and stromal cultures,^[^ ^47^, ^48^, ^49^ ^]^ the well‐known RGD peptide motif was conjugated to HA to modulate integrin‐binding (with RDG as a control).^[^ ^50^ ^]^ To vary hydrogel degradability, we used a matrix metalloproteinase (MMP)‐sensitive sequence^[^ ^49^ ^]^ to crosslink the hydrogel (modified sequence not recognizable by native MMPs as control). We encapsulated patient‐derived CAFs in three different hydrogel conditions: degradable hydrogel and integrin‐binding (D/IB), degradable hydrogel and non‐integrin‐binding (D/nIB), or non‐degradable hydrogel and integrin‐binding (nD/IB).

**Figure 3:**
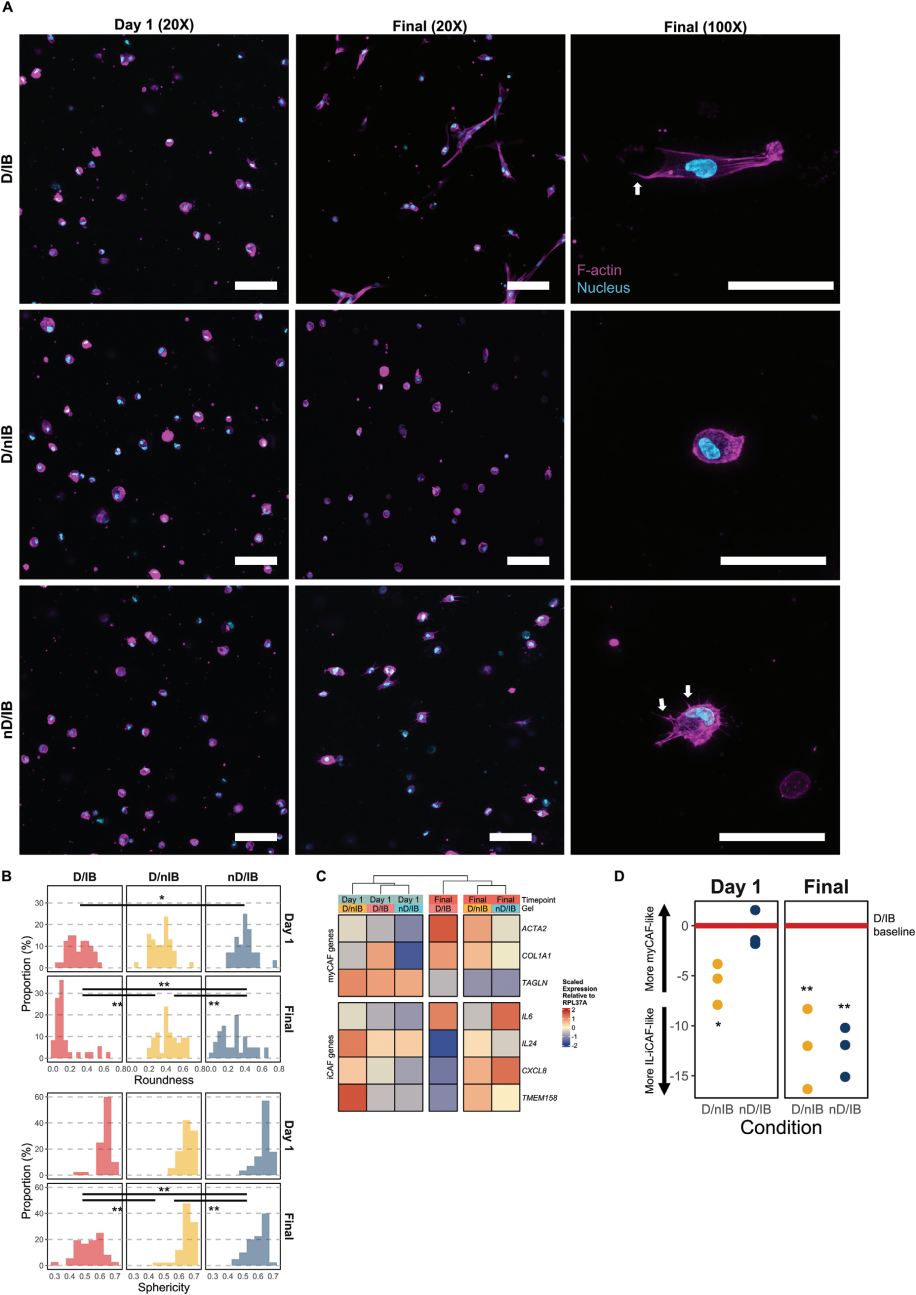
Tuning hydrogel parameters modulates CAF morphology to alter gene expression. A) Confocal images of patient‐derived CAFs cultured in various hydrogels, stained for F‐actin (phalloidin) and nuclei (DAPI): D/IB – degradable with integrin‐binding motifs (top panel); D/nIB – degradable without integrin‐binding motifs (middle panel); nD/IB – non‐degradable with integrin‐binding motifs (bottom panel). Left panel contains images of cells after 1 day in culture, while right two panels contain images of cells at the final day of culture, with the rightmost panel being images of higher magnification. White arrows point to dendritic‐like extensions. White bars represent 100 µm. (Note: final day varied across patients as CAFs took different durations to spread in D/IB gel, but was consistent for each patient. Final day was determined for each patient based once a significant majority of cells were spread). B) Quantification of cell roundness (top) and sphericity (bottom) over time for cells in each hydrogel condition (*n* = 3). Pairwise, two‐sided Wilcoxon rank‐sum test, *p < 0.05, **p < 0.01. C) Gene expression changes, as measured by RT‐qPCR, over time for cells in each hydrogel condition (*n* = 3). D) Changes in myCAF:IL‐iCAF scores at each timepoint; Pairwise, two‐sided *t*‐test, *p < 0.05, **p < 0.01.

As expected, in D/IB hydrogels, the combination of integrin‐binding motifs with MMP‐degradable sites within the hydrogel enabled encapsulated CAFs to attach to the surrounding matrix and spread (**Figure** **3**A). We observed a reduction in both cell roundness (*p* < 0.01) and sphericity (*p* < 0.01) over time, and CAFs adopted a more spindle‐like morphology (Figure 3B). High magnification images also clearly showed that these CAFs assumed a polarized phenotype with lamellopodia‐like structures. In contrast, preventing integrin‐binding maintained a spherical morphology in encapsulated CAFs (*p* = 0.76; Figure 3A,B). Notably, cells in nD/IB hydrogels could still form small protrusions, as seen by a moderate decrease mainly in roundness (*p* < 0.01) but less so in sphericity (*p* = 0.02), unlike the cells in D/IB hydrogels. Nevertheless, cells in both the D/nIB and nD/IB hydrogels were similar in that they did not adopt the polarized phenotype characteristic of cells cultured in the D/IB hydrogels. Cell spreading took up to 9 days and varied in a patient‐dependent manner, and initial experiments were performed to determine the duration for CAFs to adopt the expected morphologies (6 days for HN217P, 9 days for HN294P, and 5 days for HN338P). These identified timepoints were used for all subsequent experiments.

To investigate whether observed variations in morphology correlated with specific CAF subsets, RT‐qPCR was performed on these cultures using CAF subset‐specific markers. As shown in Figure 3C, CAFs at the start of culture were relatively similar (*p* > 0.05), except those in D/nIB hydrogels expressing higher levels of *IL24* (fold change with respect to D/IB (FC) = 3.23, *p* < 0.05) and *TMEM158* (FC = 2.68, *p* < 0.05). However, CAFs in nD/IB hydrogels became more IL‐iCAF‐like at the final timepoint as compared to CAFs in the D/IB hydrogels, as they had higher relative expression of *IL24* (FC = 6.24, p < 0.01) and *CXCL8* (FC = 4.4, *p* = 0.02). A similar trend was also observed for CAFs in D/nIB hydrogels (*IL24* – FC = 14.4, p < 0.01; *CXCL8* – FC = 3.1, *p* = 0.03). While there were no significant differences in the expression of myCAF‐related genes, we did observe that expression levels were higher for CAFs in the D/IB hydrogels at the final timepoint, when compared to CAFs in the other hydrogels (Figure 3C). We thus hypothesized that there were changes in the overall expression profile as represented by a myCAF:IL‐iCAF score (higher = myCAF‐like, lower = IL‐iCAF‐like). Analyses of these scores revealed that while CAFs encapsulated in D/nIB hydrogels were more IL‐iCAF‐like throughout culture (p = 0.01), those in D/IB hydrogels were more myCAF‐like than in the other two groups at the end of culture (both p < 0.01; Figure 3D).

### 2.4 CAF Morphology Changes Reflect Acquisition of Different CAF Subset Phenotypes

**Figure 4:**
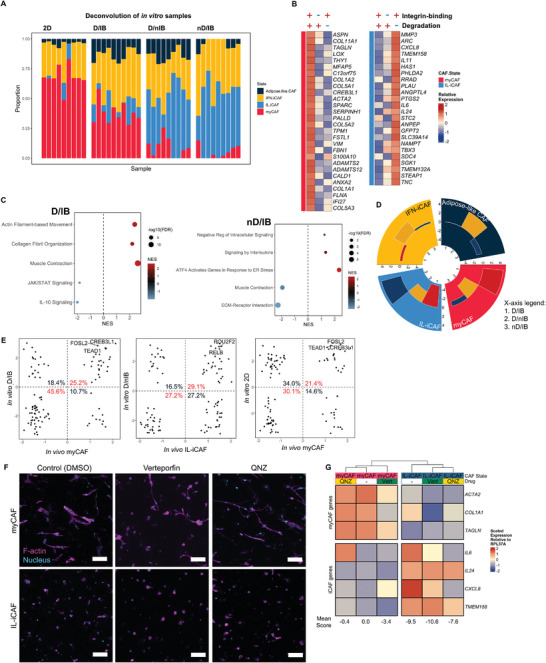
Spread spindle‐shaped cells are myCAF‐like while spherical dendritic cells are IL‐iCAF‐like. A) Deconvolution of bulk RNA sequenced CAFs that were cultured as 2D monolayers, as well as those from each hydrogel condition. Pairwise two‐sided *t*‐test was done. B) Heatmap of expression of select myCAF‐ and IL‐iCAF‐associated genes. C) Gene set enrichment analysis was performed on the DEGs of CAFs from each hydrogel, using several databases that contained sets of genes in well‐known biological pathways (see Experimental Section). CAFs cultured in the D/IB hydrogel were enriched for several myCAF‐associated pathways (left), and those in nD/IB hydrogels were enriched for several IL‐iCAF‐associated pathways (right). D) Bar plot of how each in vitro condition scored for each in vivo CAF subset, with more positive scores indicating greater similarity, and vice versa for negative scores. E) Comparing profiles of transcription factors predicted to be active in the in vitro and in vivo conditions, as computed by ARACNe‐AP. Left panel compares cells from D/IB with myCAFs, middle panel compares cells from nD/IB with IL‐iCAFs, and right panel compares 2D monolayer CAFs with myCAFs. From here on, CAFs in D/IB and nD/IB are referred to as in vitro myCAFs and IL‐iCAFs respectively. F) Confocal images of CAFs treated with the respective drug, stained for F‐actin (phalloidin) and nuclei (DAPI). White bars represent 100 µm. G) Changes in gene expression, as measured by RT‐qPCR, upon treatment of in vitro myCAFs and IL‐iCAFs with verteporfin (YAP/TEAD inhibitor) and QNZ (NF‐κB inhibitor). Mean myCAF:IL‐iCAF scores are found below. Pairwise two‐sided t‐test was performed.

To confirm these at a global level, we subjected CAFs cultured in the different hydrogel conditions, as well as those cultured on plastic as monolayers (2D CAFs), to bulk RNAseq. We used CibersortX^[^ ^51^ ^]^ to deconvolute the data to decipher the relative proportions of CAF subsets within 2D CAFs. myCAFs dominated 2D cultures (68.0 ± 9.4%; data presented as mean ± standard deviation (SD)) in line with the observation that CAFs cultured as monolayers adopt a spindle‐like morphology (**Figure** **4**A). Following encapsulation of these 2D‐cultured CAFs into the three hydrogel conditions, we found that while all cultures contained a mix of 2 to 4 CAF subsets, there were significant variations in the proportions of each CAF subset (Figure 4A). D/IB hydrogels supported the highest proportion of myCAFs (38.0 ± 9.2%, *p* < 0.01) and the lowest proportion of IL‐iCAFs (10.2 ± 9.4%, *p* < 0.01) as compared to the D/nIB and nD/IB hydrogel groups, corroborating previous RT‐qPCR results. On the other hand, D/nIB and nD/IB hydrogel conditions supported higher proportions of IL‐iCAFs (44.9 ± 17.8% and 51.2 ± 15.0%, respectively; both *p* < 0.01) and lower proportions of myCAFs (9.3 ± 8.4% and 4.9 ± 5.4%, respectively; both *p* < 0.01) as compared to the D/IB hydrogel group.

Furthermore, CAFs in the nD/IB hydrogel group had higher expression of IL‐iCAF‐ associated genes compared to those in the D/nIB hydrogel group (Figure 4B; Table S6, Supporting Information), suggesting that the former condition is more conducive for IL‐iCAF generation. Similar biological pathways were therefore also enriched in these in vitro CAFs when compared to their in vivo counterparts: actomyosin structure organization for CAFs in D/IB hydrogels, and signaling by interleukins for CAFs in nD/IB hydrogels (Figure 4C). Lastly, we performed GSEA using differentially expressed gene sets derived from scRNAseq, to compute a correlation score between the cultured CAFs and the in vivo CAF subsets (see Experimental Section). Allowing CAFs to spread (D/IB) induced a strong myCAF phenotype, while dendritic and spherical CAFs in the non‐degradable hydrogel (nD/IB) were IL‐iCAF‐like (Figure 4D). In contrast, round and spherical CAFs in D/nIB were neither CAF subset‐specific nor myCAF‐ or IL‐iCAF‐like.

We next asked if similar TFs were active in the CAF subsets derived using the different hydrogel conditions, as previously described in the scRNAseq data. Using ARACNe‐AP and GSEA, we identified TFs that were associated with gene signature of CAFs in each hydrogel condition. Comparing these with those identified in Figure 2, we observed a 70.8% concordance between CAFs in D/IB hydrogels and in vivo myCAFs (Figure 4E). This included in vivo TFs such as *FOSL2*, *CREB3L1*, and *TEAD1*. The similarity between the in vivo IL‐iCAFs and CAFs in nD/IB gels was lower at 56.3% but still identified *RELB* and *POU2F2* as key TFs. Importantly, while 2D monolayer CAFs exhibit myofibroblastic features,^[^ ^15^ ^]^ our results showed that their similarity in TF activities to in vivo myCAFs was only at 51.5%, lower than that of the D/IB hydrogel‐cultured CAFs. The full list of TF enrichment scores can be found in Table S7 (Supporting Information).

To rule out previously reported findings that stiffer hydrogels induce iCAF‐like gene expression and vice versa,^[^ ^13^ ^]^ we further characterized the biophysical characteristics of the hydrogels over time. As expected, all hydrogels were initially similar in stiffness. However, only the D/nIB gels became significantly less stiff over time (Figure S1A, Supporting Information). We also saw no significant differences in swelling ratio (Figure S1B, Supporting Information), suggesting that hydrogel porosity was similar across groups. These data suggested that cell spreading was the main driver of the observed myCAF‐IL‐iCAF dichotomy instead of hydrogel stiffness. Taken together, we concluded that spread CAFs in D/IB gels were myCAF‐like, while dendritic and spherical CAFs in nD/IB gels were IL‐iCAF‐like.

### 2.5 TEAD and NF‐κB may Partially Regulate the myCAF and IL‐iCAF Phenotypes

We next asked whether specific TF‐inhibiting drugs could be used in these cultures to target CAF subsets, and identified verteporfin, which targets YAP/TEAD, and QNZ, which targets NF‐κB (viability assays indicated that all drugs used in this study resulted in < 30% cell death, see Figure S2A, Supporting Information). Morphological analysis of verteporfin‐ or QNZ‐treated cultures revealed that verteporfin induced a population of in vitro myCAFs to become rounder (FC with respect to control = 1.82, *p* = 0.01) and more spherical (FC = 1.13, *p* = 0.02; Figure 4F and Figure S2B, Supporting Information). This was associated with a decreasing trend of myCAF‐associated gene expression in in vitro myCAFs (*ACTA2* FC = 0.73; *COL1A1* FC = 0.49; *TAGLN* FC = 0.78), although the changes were not statistically significant (Figure 4G). In contrast, QNZ may induce a lower, but not statistically significant, expression of some IL‐iCAF markers in in vitro IL‐iCAFs, such as *IL6* (FC with respect to nD/IB control = 0.11) and *CXCL8* (FC = 0.08; Figure 4G). Overall, gene expression profile scoring yielded similar results for both verteporfin‐treated myCAFs and QNZ‐treated IL‐iCAFs when compared to their respective controls. Hence, TEAD and NF‐κB are likely not the sole regulators of gene expression in myCAFs and IL‐iCAFs but certainly appear to have a functional consequence on CAF plasticity.

### 2.6 Microtubule Dynamics are Responsible for Switch from myCAF to IL‐iCAF

**Figure 5:**
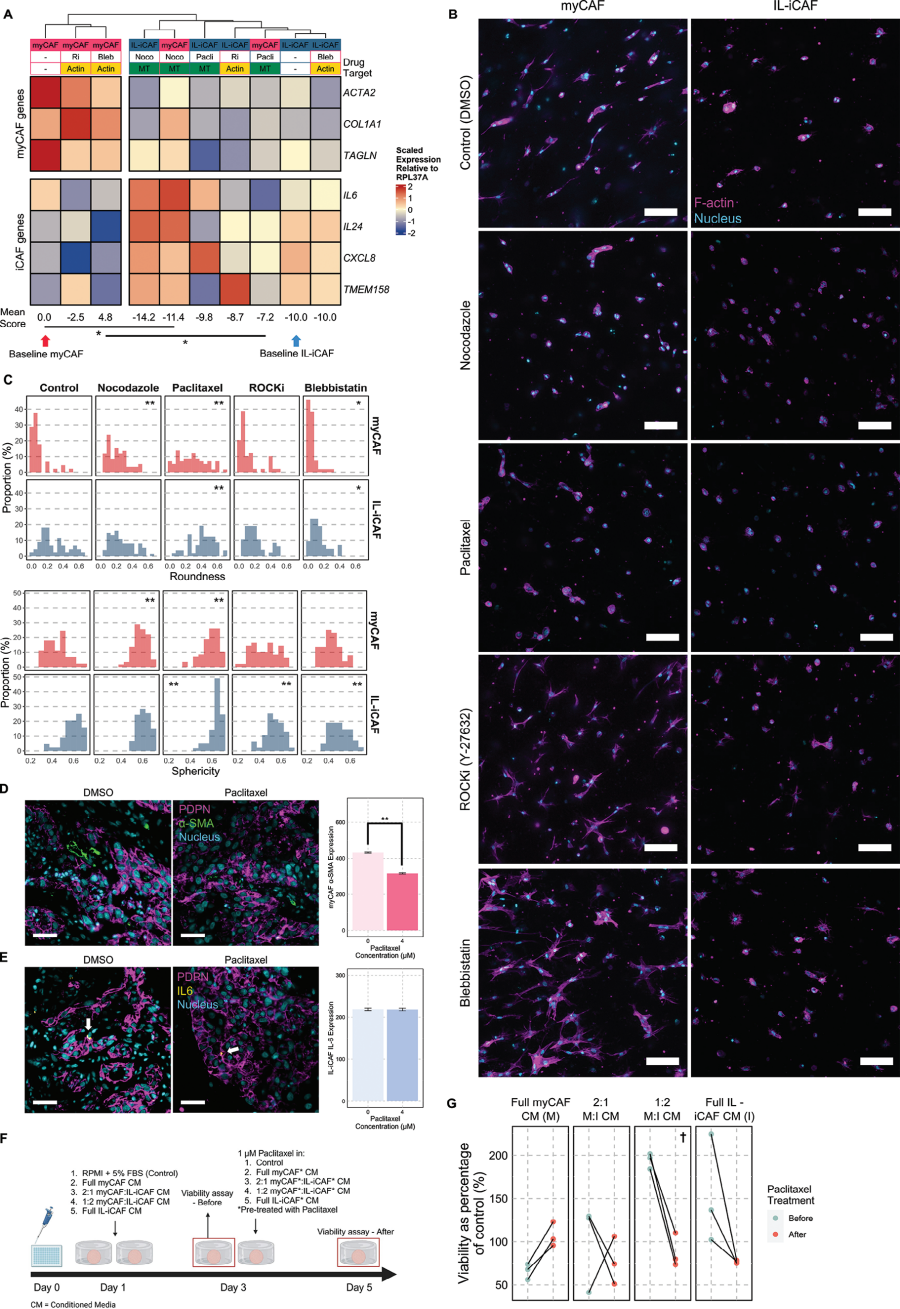
Microtubule dynamics contribute to switching between myCAF‐ and IL‐iCAF‐like gene expression. A) Heatmap of changes in gene expression for in vitro myCAFs and IL‐iCAFs treated with cytoskeleton‐targeting drugs, as measured by RT‐qPCR, with corresponding mean myCAF:IL‐iCAF score below (*n* = 3). Arrows below point to respective baselines. Pairwise, two‐sided *t*‐test, *p < 0.05. B) Confocal images of CAFs treated with the respective drug, stained for F‐actin (phalloidin) and nuclei (DAPI). White bars represent 100 µm. C) Quantification of cell roundness and sphericity of CAFs treated with the respective drug (*n* = 3). Pairwise, two‐sided Wilcoxon rank‐sum test, *p < 0.05, **p < 0.01 when compared to respective controls. D,E) Fluorescence images of tumor explants, treated with paclitaxel or not, stained for D) PDPN and α‐SMA and (E) PDPN and IL‐6, as well as the quantification for their respective expression of α‐SMA (*n* = 4056 cells) and IL‐6 (*n* = 2276 cells). Pairwise, two‐sided Wilcoxon rank‐sum test, **p < 0.01. White arrows on E) point to location of IL‐6 signal. White bars represent 100 µm. F) Schematic of how cancer spheroids were educated and treated with paclitaxel. Untreated CAFs had an equivalent amount of DMSO added before collection of conditioned media, as a control for the paclitaxel‐treated CAFs. FBS = fetal bovine serum; CM = conditioned media. (G) Viability of cancer spheroids when pre‐conditioned with media from myCAF and IL‐iCAF in varying proportions, then treated with 1 µM paclitaxel, in the presence of conditioned media from paclitaxel‐treated myCAF and IL‐iCAF in matched proportions (n = 3, pairwise paired two‐sided t‐test performed on percentage change from control; ^†^p < 0.1).

Given the association between morphology with adoption of myCAF or IL‐iCAF phenotypes, we asked whether microtubule dynamics and/or actomyosin contractility contribute to the maintenance of these CAF subsets in vitro. We observed that actomyosin‐targeting drugs (ROCK inhibitor (ROCKi) and blebbistatin) did not alter the gene expression profiles of in vitro myCAFs and IL‐iCAFs. However, microtubule‐targeting drugs, particularly nocodazole (*p* < 0.05), significantly induced a more IL‐iCAF‐like phenotype in in vitro myCAFs (**Figure** **5**A). Furthermore, the significant changes in myCAF to IL‐iCAF gene expression profiles were correlated with increased roundness and sphericity (Figure 5B,C). Nocodazole and paclitaxel caused almost all cells, both in vitro myCAFs and IL‐iCAFs, to become rounder and more spherical compared to the control (all *p* < 0.01, except nocodazole on IL‐iCAFs where no significant change was detected). The effect observed here was also greater than that induced by verteporfin (roundness FC = 1.82, sphericity FC = 1.13), as roundness (nocodazole FC = 2.49, paclitaxel FC = 3.28) and sphericity (nocodazole FC = 1.30, paclitaxel FC = 1.31) both increased to larger extents. ROCKi and blebbistatin had no effect on in vitro myCAFs, which remained dendritic‐like and spread (*p* > 0.05, except slight reduction in roundness when treated with blebbistatin, *p* = 0.04). The actomyosin‐targeting drugs also slightly reduced sphericity in in vitro IL‐iCAFs (both *p* < 0.01), while blebbistatin reduced roundness (*p* < 0.01), but cells were still not as spread as the in vitro myCAFs (both *p* < 0.05).

Given that paclitaxel is used to treat HNSCC patients^[^ ^52^ ^]^ and the observed shift from the myCAF to IL‐iCAF phenotype after paclitaxel‐treatment of myCAFs, we sought to validate these results using an ex vivo patient‐derived tumor explant model. We recently showed that tumor explants retain both myCAF and iCAF subpopulations in HNSCC and are suitable for evaluating drugs targeting the tumor microenvironment.^[^ ^53^ ^]^ We exposed these tumor explants to either paclitaxel or DMSO (control), and subsequently probed for PDPN (pan‐CAF marker in HNSCC^[^ ^31^ ^]^) and either α‐SMA (*ACTA2*) for myCAFs or IL‐6 for IL‐iCAFs. Using QuPath's cell detection algorithm, expression of these markers could be quantified at the single‐cell level (see Figure S2C,D, Supporting Information for thresholds used to identify myCAFs and IL‐iCAFs). The results showed that changes in the ex vivo myCAFs mirrored the in vitro RT‐qPCR results (Figure 5A,D,E). When treated with paclitaxel, both the in vitro myCAFs and the PDPN^+^α‐SMA^+^ myCAFs displayed a reduction in *ACTA2* and α‐SMA (median percentage change with treatment = +7.7%, p < 0.01) expression, respectively. These changes were also associated with a loss of polarization in the in vitro myCAFs cultured in the D/IB hydrogel (Figure S2E, Supporting Information). However, while paclitaxel induced a slight increase in *IL6* expression in the in vitro IL‐iCAFs, PDPN^+^IL‐6^+^ IL‐iCAFs did not show significant changes in IL‐6 expression (median percentage change with treatment = −1.7%, *p* = 0.91).

### 2.7 Effect of myCAFs and IL‐iCAFs on Microtubule‐Targeting Anti‐Cancer Drug Response

Given the differences in response of myCAFs and IL‐iCAFs to microtubule‐targeting drugs, we hypothesized that they would differentially influence cancer cell drug sensitivity. To test this using our CAF cultures, we first applied conditioned media from myCAFs and IL‐iCAFs to pre‐educate cancer cells grown as spheroids. Conditioned medium from paclitaxel‐treated myCAFs and/or IL‐iCAFs were then applied to the cancer spheroids in the presence of paclitaxel to recapitulate the treatment of tumors with paclitaxel in vivo. To address the varying proportions of different CAF subsets in patients, we also mixed the media in several ratios to elucidate any compound effects (see Figure 5F for a schematic). Interestingly, cancer spheroids may become more sensitive to paclitaxel only when myCAF and IL‐iCAF conditioned media were mixed in a 1:2 ratio (viability as a percentage of control decreased from 194% to 88%, *p* = 0.06; Figure 5G; Figure S2F (Supporting Information) contains a representative image of the spheroids used for this experiment).

Hence, we asked if these findings could be observed in the clinic. Using data from The Cancer Genome Atlas,^[^ ^54^ ^]^ we identified HNSCC patients who received therapies targeting microtubules (paclitaxel or docetaxel). Then, using bulk RNAseq data, we stratified patients according to their relative abundance of myCAFs and IL‐iCAFs as determined by single sample GSEA. Kaplan‐Meier analysis showed that the relative abundance of myCAF and IL‐iCAF, but not their individual abundances, impacted overall survival among patients treated with paclitaxel or docetaxel (Figure S3A, Supporting Information). Specifically, lower myCAF:IL‐iCAF abundance was the worst prognostic for HNSCC patients. On the other hand, an intermediate myCAF:IL‐iCAF abundance was the best prognostic, which was reflected by reduced cancer spheroid viability when in the presence of 1:2 myCAF:IL‐iCAF conditioned media (Figure 5G). When the same analysis for HNSCC patients not treated with paclitaxel or docetaxel was repeated, we found that there was no correlation between the two CAF states and overall survival (Figure S3B, Supporting Information). Taken together, these results suggest that the benefits of paclitaxel and docetaxel could be partially mediated by inducing a more IL‐iCAF‐like state in the myCAFs, but only if there were some IL‐iCAFs present to begin with. These findings therefore highlight that microtubule dynamics may play an important role in the switch from myCAFs to IL‐iCAFs; and targeting microtubules could potentially benefit patients with an intermediate myCAF:IL‐iCAF abundance.

## 3 Discussion

Despite our increased understanding of CAF heterogeneity, the functional roles of CAF subpopulations remain largely unknown and require further investigation.^[^ ^55^ ^]^ This is due in part to a lack of physiologically relevant models reflecting CAF diversity for such studies. In vitro monolayer culture of CAFs typically promotes the myCAF phenotype only,^[^ ^15^ ^]^ and cultured cells typically lose the expression of typical CAF activation markers and ligands such as *IL6* and *HGF*.^[^ ^31^ ^]^ Here, we asked if a customizable hydrogel system could be optimized to generate models of different CAF subsets by incorporating microenvironmental features. In vivo transcriptomic data, specifically, transcriptional activities and predicted ligand‐receptor interactions from scRNAseq data of HNSCC patients, suggested differences in the myCAF and IL‐iCAF microenvironments which we were able to partially incorporate into a tunable hydrogel system. This approach yielded the derivation of both myCAF and IL‐iCAF subsets from the same pool of 2D‐expanded and then hydrogel‐encapsulated patient‐derived CAFs, without the need for explant culture and cell sorting, as was previously reported to be necessary for the culture of these CAF subsets.^[^ ^14^ ^]^

Previous studies have demonstrated how hydrogel‐induced changes in cell morphology can influence encapsulated cells to adopt different phenotypes.^[^ ^18^, ^56^, ^57^ ^]^ Cao and colleagues recently showed how hydrogel stiffness mediates the generation of different CAF subsets – while myCAFs were generated in softer matrices that enabled cell spreading, iCAFs were derived from culture in stiffer matrices that prevented cell spreading.^[^ ^13^ ^]^ On the contrary, while similar correlations between cell shape and phenotype were made in our study, we found that myCAFs and iCAFs can be generated using hydrogels of similar initial stiffness values, suggesting that the myCAF‐iCAF switch is not merely regulated by hydrogel stiffness. As shown in this study, TF and CCN analyses supported the role of high cellular tension for myCAF culture, while the contrary could potentially generate iCAFs. Our corresponding finding that modulating hydrogel degradation in the presence of integrin ligands to control cell spreading indeed induced encapsulated CAFs to adopt myCAF or iCAF states, suggests that cellular tension likely plays a fundamental role in controlling CAF plasticity. Work is ongoing in our laboratory to leverage this parameter to generate CAF heterogeneity in other bioengineered culture systems.

The ability to culture myCAFs and iCAFs in vitro from the same initial source opens up opportunities to evaluate the effect of different treatment modalities (cytotoxic chemotherapy, radiation therapy, or targeted therapies) on CAF diversity. In this study, we discovered that microtubule inhibition may affect myCAF populations, switching myCAFs to an IL‐iCAF‐like state. Given that iCAFs are immunomodulatory, it would be interesting to investigate whether microtubule‐inhibiting drugs could augment the efficacy of immune checkpoint inhibitors. This approach may be useful as the combination of paclitaxel and immunotherapies is already being explored in the clinic.^[^ ^58^ ^]^ Our findings also highlight the need to study the effect of cancer treatment on CAF diversity (and hence treatment outcome), especially in tumors with high CAF content.

There are a few limitations to this study. First, the hydrogels used here were limited to the fibronectin‐derived RGD peptide. The role of other microenvironmental components remains unexplored and is ongoing work. Secondly, we have not investigated the universality of this approach for CAFs of other cancer types. This would require analyzing the scRNAseq datasets of patient tumors from other cancer types to determine if cellular and microenvironmental features of different CAF subsets are similar to that in HNSCC. Lastly, the CAF subsets generated in this study are cultured in different hydrogel matrices. Given the importance of relative abundances of CAF subsets (Figure 5G; Figure S3, Supporting Information), it would be important to eventually incorporate myCAFs and iCAFs within the same matrix for co‐culture with cancer cells, as is the case in a typical TME.

In conclusion, using in vivo transcriptomic data to guide the selection of hydrogel parameters,^[^ ^59^ ^]^ we established in vitro cultures of different CAF subsets in HNSCC using a bioengineered hydrogel. These in vitro myCAF and IL‐iCAF cultures revealed useful insights, such as the effect of microtubule‐targeting standard‐of‐care drugs on different CAF subsets. With growing interest in targeting CAF heterogeneity, and their roles in mediating cancer progression and drug resistance, these bioengineered models of CAF subsets may serve as robust tools for granular understanding of CAF subset functions, and may also be useful for the screening of CAF‐targeting therapeutics.

## 4 Experimental Section

### scRNAseq Data and Data Processing

Raw counts data from tumors (10 from primary site and 7 from metastatic site) of 10 human papillomavirus negative (HPV^−^) HNSCC patients were obtained from the Cancer Therapeutics Research Laboratory at the National Cancer Centre of Singapore (co‐headed by NG Iyer). scRNAseq data were obtained from previosuly published studies.^[^ ^53^, ^67^ ^]^ All raw counts data will be available for access (refer to Data Availability Statement).

scRNAseq data was preprocessed using Cellranger (10x Genomics, version 6.1.2) with default parameters, and aligned with the to the GRCh38 reference genome. We then used the SCTransform method of the Seurat package version 4.3.0.1^[^ ^68^, ^69^ ^]^ for subsequent processing. Cells were filtered for unique molecular identifier counts (200 – 50 000), feature counts (100 – 6000), and percentage of mitochondrial gene expression (< 15%); while genes were filtered to keep only those that were expressed by at least 3 cells. We then used the Harmony package version 0.1.1 to integrate the data.^[^ ^70^ ^]^ Upon Uniform Manifold Approximation and Projection (UMAP) for dimensionality reduction, unsupervised Louvain clustering was performed. Differentially expressed genes (DEGs) across clusters were computed using the Wilcoxon rank sum test. To determine the number of CAF clusters, we used the singleCellHaystack algorithm version 1.0.0.^[^ ^20^ ^]^

### Gene Set Enrichment Analysis (GSEA)

We used clusterProfiler's (version 4.4.4)^[^ ^71^ ^]^ implementation of GSEA to annotate cell types, determine enriched biological pathways, and compare in vitro and in vivo gene signature and transcription factor activity profiles. Cell type and biological pathway gene sets were obtained from the Molecular Signatures Database (mSigDB).^[^ ^72^ ^]^ CAF subset gene signatures were obtained from the studies published by Chung,^[^ ^24^ ^]^ Dominguez,^[^ ^25^ ^]^ Elyada,^[^ ^26^ ^]^ Givel,^[^ ^27^ ^]^ Hanley,^[^ ^28^ ^]^ Kieffer,^[^ ^14^ ^]^ Puram,^[^ ^31^ ^]^ Wang,^[^ ^29^ ^]^ Wu,^[^ ^30^ ^]^ Zhu,^[^ ^32^ ^]^ and their respective colleagues. When comparing the in vitro to in vivo CAFs, a score was computed for cells in each hydrogel condition. Briefly, for every pair of hydrogel condition and CAF subset, we separately computed each hydrogel condition's NES for the significantly up‐ and downregulated genes by each CAF subset (|log2 fold change| > 0.25, *p* value < 0.05, and identified by corresponding singleCellHaystack gene set). The CAF subset score was then defined as the difference between the former and latter NES. This was to ensure that each in vitro condition was similar to the in vivo CAF subset in both the up‐ and downregulated gene signatures.

For single sample GSEA in stratifying HNSCC patients from TCGA, we used the R‐based guided user interface from mSigDB (ssGSEA version 2.0).^[^ ^73^ ^]^ CAF subset gene sets were obtained by DEG analysis comparing each CAF subset to all other cells in the tumor, and filtered by log2 fold change (> 0.25 or < −0.25) and adjusted p value (< 0.05). CAF subset scores for each patient were calculated similarly for the previously described CAF subset score. We then defined relative abundance of 2 CAF subsets as the difference between 2 CAF subset scores.

### Transcription Factor (TF) Activity and Ligand‐Receptor Interaction (LRI) Analyses

To conduct TF activity analysis, pySCENIC version 0.12.1^[^ ^36^ ^]^ and ARACNe‐AP^[^ ^37^ ^]^ analyses were run on the command line interface, using the curated human transcription factor list defined by pySCENIC. We made use of Seurat^[^ ^74^ ^]^ to process the pySCENIC output, including dimensionality reduction and clustering, with batch correction done using Harmony.^[^ ^70^ ^]^ The Slingshot version 2.4.0 algorithm^[^ ^38^ ^]^ aided in predicting pseudotime trajectory. ARACNe‐AP was performed with 10 and 100 bootstraps for single cell and bulk RNAseq data, respectively.

For LRI analysis, CellPhoneDB version 4.1.0^[^ ^44^ ^]^ and NATMI^[^ ^45^ ^]^ were run on the command line interface, while NicheNet‐dependent Scriabin version 0.0.0.9000^[^ ^45^, ^46^, ^75^ ^]^ was run on R. As Scriabin offered multiple LR databases, we selected ConnectomeDB2020. Furthermore, given the single cell resolution of Scriabin, we adhered to the recommendation of running the algorithm separately for each patient. A LRI was defined to be significant for each CAF subset if it was detected in at least 3 patients. LRIs detected by Scriabin and CellPhoneDB also had to be statistically significant (p < 0.05) for them to be considered in subsequent analyses. Unlike CellPhoneDB and Scriabin, NATMI did not offer statistical significance for LRIs. Hence, LRIs computed by NATMI were first ranked by the geometric mean of each interaction's weight and specificity, with the top 20% for each receiving cell type taken to be “true” interactions.

### Cell Culture and Hydrogel Fabrication

All patient‐derived cells were obtained in strict compliance with the Singhealth Centralized Institutional Review Board ethical guidelines, with written consent for research use (Singhealth Centralized Institutional Review Board (CIRB) 2014–2093). In vitro experiments in this paper used cells from 3 patients, HN217, HN294, and HN338. CAFs from the core of tumors were isolated via differential trypsinization (Thermo Fisher Scientific, USA) to separate them from cancer cells, and confirmed by their typical spindle shape. They were grown in Roswell Park Memorial Institute (RPMI) 1640 media (Thermo Fisher Scientific, USA) supplemented with fetal bovine serum (10%, Cytiva, USA), and at 37 °C and 5% CO_2_. We initially cultured the CAFs with an additional penicillin‐streptomycin (1%, Thermo Fisher Scientific, USA), which was removed after a few passages. Media changes occurred every 1 – 2 times a week. For drug treatment studies, we ensured that they were not excessively toxic to cells (> 70% viability as measured by CellTiter‐Glo (Promega, USA) on the FLUOstar OPTIMA (BMG Labtech, Germany); Figure S2, Supporting Information). In particular, we used verteporfin (1 µM, Tocris Bioscience, UK), QNZ (10 µM, Abcam, UK), nocodazole (1 µM, Abcam, UK), paclitaxel (4 µM, Thermo Fisher Scientific, USA), Y‐27632 (50 µM, Stem Cell Technologies, Canada), and blebbistatin (20 µM, Selleckchem, USA). We initiated drug treatment only when the respective states were attained, and for 2 days in the dark.

The protocol for HA hydrogel fabrication was adapted from past work.^[^ ^49^ ^]^ Briefly, every hydrogel contained a mixture of 0.7% thiolated HA (Advanced Biomatrix, USA), mono‐SVA‐PEG‐acrylated integrin‐binding peptide (17.6 mg mL^−1^), di‐SVA‐PEG‐acrylated crosslinker (3.3 mg mL^−1^), and sodium hydroxide (1.3 mM, NaOH; Thermo Fisher Scientific, USA) in phosphate buffered solution (PBS; Thermo Fisher Scientific, USA). We purchased PEG‐acrylate (MW 3400) from Laysan Bio (USA), and peptides from GenScript (USA). The addition of NaOH was required to initiate the addition reaction between thiol and acrylate groups. The integrin‐binding peptide had an amide‐G**RGD**S sequence (RGD), while the scrambled version to prevent integrin‐binding had an amide‐G**RDG**S sequence (RDG). For crosslinking, we utilized a matrix metalloproteinase (MMP)‐degradable peptide sequence, KGGGPQG **I** WGQGK‐acetyl (PQ)^[^ ^49^ ^]^; and to prevent degradation, the sequence was altered to replace L‐isoleucine with D‐isoleucine (isoPQ), which is not recognized by native MMPs. CAFs were encapsulated in the gel at a density of 1 × 10^5^ cells in a 36 µL gel and cultured up to 6, 9, and 5 days for CAFs from HN217, HN294, and HN338, respectively. To ensure uniformity in hydrogel shape, custom 2 mm‐thick polydimethylsiloxane molds (PDMS; SYLGARD 184 Silicone Elastomer from The Dow Chemical Company, USA) were placed on glass slides. The PDMS molds also contained punched 5 mm‐wide holes into which we could pipette and culture the hydrogels for the entire duration. We used the standard formula to fabricate these PDMS molds, using a 10:1 polymer:crosslinker mass ratio, which we mixed well and degassed, then crosslinked at 60 °C for 1 h.

### Biophysical Characterization of Hydrogels

Storage moduli and loss factors were measured using an AR‐G2 rheometer (TA Instruments, USA) with an 8 mm parallel plate geometry. The protocol was developed in reference to that reported by Zuidema and colleagues.^[^ ^76^ ^]^ Specifically, we conducted time sweeps using a strain of 0.05% strain and a frequency of 0.1 Hz. In these experiments, gel dimensions, including volume and cell numbers, were scaled 1.5 times (1.5 × 10^5^ cells in each 54 µL gel) in an 8 mm PDMS mold instead of a 5 mm one. Gels were also pre‐fixed with 4% paraformaldehyde (PFA; Sigma‐Aldrich, USA) for 30 min at room temperature, followed by washing with PBS. As for swelling ratio, the gels were also fixed under the same conditions, gently dabbed them with filter paper to remove excess moisture, then weighed for their wet mass. Following which, they were frozen at −80 °C, lyophilized, and weighed for their dry mass. The swelling ratio was then calculated as the ratio of the difference between wet and dry mass to the dry mass.

### Fluorescence Microscopy of Hydrogel‐Cultured Cells and Morphology Analysis

All staining steps were carried out with gentle shaking at room temperature, washing with PBS for 10 min, unless otherwise stated. Cells were fixed in situ with 4% PFA for 30 min, washed, and permeabilized with Triton‐X 100 (0.5%, Nacalai Tesque, Japan) for 30 min. After which, gels were then washed again. We stained for F‐actin and nuclei with CF633‐conjugated phalloidin (0.67 U mL^−1^, Biotium, USA) and DAPI (4 µM, Abcam, UK), respectively. This step was carried out at 4 °C overnight, and gels were subsequently washed five times.

Imaging was carried out using the Olympus FV3000 (Japan). The method for cell segmentation was adapted from the one developed by Bajcsy and colleagues,^[^ ^77^ ^]^ with the addition of a region growing step to better identify dendritic protrusions. Briefly, we despeckled, filtered, and binarized the images. Then, hole filling, image opening, image closing, and a second despeckling were performed. This image was used a seed for the subsequent region growing algorithm, with the despeckled image as a reference. Thereafter, with manual inspection, we only utilized cells that met the following 3 criteria for quantification: 1) contained a nucleus, 2) not cut off by the edges, and 3) detected cell shape matched original image. The 3D objects counter in Fiji provided measurements of cell volume and surface area, which we used to calculate sphericity as a measure of how spindle‐like a cell is [((π × (6 × Volume)^2^)^1/3^) ÷ (Surface Area)]. To measure the degree of dendritic‐like protrusions, we used the Roundness 2D 3D function (6 × Volume / (Diameter^3^ × π)) of the Xlib plugin.^[^ ^78^ ^]^ A Fiji plugin is available for download (refer to Data Availability Statement).

### RNA Extraction and Real Time Quantitative Polymerase Chain Reaction (RT‐qPCR)

Cells were first extracted from the hydrogel before RNA extraction. Briefly, hydrogels were mechanically dissociated with a micropipette and incubated with hyaluronidase (2 mg mL^−1^, dissolved in PBS) and TCEP (20 mm, tris(2‐carboxyethyl)phosphine; Sigma‐Aldrich, USA) for 45 min at 37 °C. We then washed cells twice with PBS and centrifuged them at 800 x g for 10 min after the first wash, and at 500 x g for 3 min after the second. RNA extraction was performed using the RNeasy Plus Mini or Micro Kits (QIAGEN, Germany). If RT‐qPCR was to be carried out, reverse transcription was performed using iScript Supermix (Bio‐Rad, USA) according to the manufacturer's protocol. The RT‐qPCR reaction was then set up using SYBR Green (Bio‐Rad, USA) with a 15 µL reaction containing 5 ng complementary DNA and 0.33 µm for each forward and reverse primer. The reaction was carried out on the QuantStudio 5 system (Thermo Fisher Scientific, USA). Primers used, which were purchased from Integrated DNA Technologies (USA), can be found in **Table** **1**.

**Table 1:** Sequences (5′‐3′) of primers used to carry out RT‐qPCR.

Gene	Forward	Reverse
*ACTA2*	CTGGACTCTGGAGATGGTGT	TCTCACGCTCAGCAGTAGTA
*COL1A1*	CATGGAGACTGGTGAGACCT	GCCATACTCGAACTGGAATC
*TAGLN*	AGTGCAGTCCAAAATCGAGAAG	CTTGCTCAGAATCACGCCAT
*IL6*	ACCGGGAACGAAAGAGAAGCTC	AGAAGGCAACTGGACCGAAGG
*IL24*	TTGCCTGGGTTTTACCCTGC	AAGGCTTCCCACAGTTTCTGG
*CXCL8*	GCAGCTCTGTGTGAAGGTGCAGTT	TCTGTGTTGGCGCAGTGTGGTC
*TMEM158*	CGGTGTGCTTCGTGCTGTAG	GGAAGCGCAGCACAAACCTT
*RPL37A*	ATTGAAATCAGCCAGCACGC	AGGAACCACAGTGCCAGATCC

To compare gene expression profiles, the myCAF:IL‐iCAF scores were calculated such that higher scores represented a more myCAF‐like profile, and lower scores indicated a more IL‐iCAF‐like phenotype (Equation 1). The scores were then normalized to the respective baseline conditions (D/IB or D/IB without drugs, where applicable), through a difference in scores.

(1)

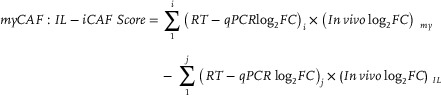




RT‐qPCR log_2_FC = log_2_(fold change) of cycle threshold of gene relative to that of *RPL37A*;

In vivo log_2_FC_my/IL_ = log_2_(fold change) of gene i/j for myCAF/IL‐iCAF obtained from scRNAseq;

i = myCAF‐associated gene; j = IL‐iCAF‐associated gene

### Bulk RNA Sequencing and Analysis

The sequencing was outsourced to the Duke‐NUS Genome Biology Facility (DGBF). Before sequencing, RNA integrity was first determined with the Agilent RNA 6000 Nano Kit on the Agilent 2100 Bioanalyzer System (both from Agilent Technologies, USA). They then performed library preparation using the Illumina Truseq Stranded mRNA library, and sequencing using the Illumina Hi‐Seq platform with 150‐bp paired‐end reads (both from Illumina, USA). The STAR version 2.7.9a algorithm was used to align sequencing reads and predict gene counts (sjdbOverhang = 149), with the hg38 reference transcriptome.^[^ ^79^ ^]^ For heatmap plotting, we used ComBat‐seq (sva version 3.44.0) to correct for batch effects,^[^ ^80^ ^]^ and plotted the scaled, mean, arcsinh‐normalized counts per million values. DEGs were determined with DESeq2 version 1.36.0,^[^ ^81^ ^]^ with sequencing batch included in the model; and log2 fold change shrinkage was performed using apeglm version 1.18.0.^[^ ^82^ ^]^ As we wanted to predict the proportions of CAF subsets in each bulk sample, deconvolution was done using CibersortX.^[^ ^83^ ^]^ The signature matrix was generated using the scRNAseq counts, with sampling set to 0.9. All other parameters were set to the recommended values.

### Tumor Explant Culture, Immunostaining, and Analysis

Patient explants were sourced in strict compliance with the National Cancer Centre Singapore's ethical guidelines, with written consent for research use (CIRB 2014/2093) The protocol for explant culture was adapted from a previously published work.^[^ ^53^ ^]^ Briefly, upon excision from the patient, tumor samples were precision‐cut to 300 µm in thickness and layered on a similar HA hydrogel (1.12% HA, 12.3 mg mL^−1^ RGD, and 6.6 mg mL^−1^ PQ) used to culture the CAFs. One patient tumor explant was used in this experiment (HN440). After letting the explants recover for 24 h, they were then cultured with either paclitaxel (4 µm) or an equivalent amount of DMSO for 48 h. Upon which, the tumor explants were fixed with neutral buffered formalin (Sigma‐Aldrich, USA) for 48 h and then embedded in paraffin using standard dehydration protocols. The paraffin‐embedded explants were then sliced to 5 µm sections for immunostaining using a microtome.

For immunostaining, paraffin was removed from sections using xylene (thrice for 2 min each), then progressively rehydrated, for 2 min each, in 100% ethanol twice, 95% ethanol once, 70% ethanol once, and tap water twice. Next, they were permeabilized with Triton‐X 100 (0.5%, Nacalai Tesque, Japan). Antigen retrieval was performed using a citrate buffer (10 mm, sodium citrate and citric acid from Sigma‐Aldrich, USA) at sub‐boiling temperatures (95–98 °C) for 20 min. After cooling for 30 min, sections were washed thrice in pH 7.6 Tris‐buffered saline with Tween‐20 (Tris and NaCl from Sigma‐Aldrich, USA, Tween‐20 from Nacalai Tesque, Japan). Blocking was performed with 5% bovine albumin serum for 1 h, and the primary antibodies were incubated overnight at 4 °C (anti‐PDPN from Dako, Denmark, catalog M3619, 1:50 dilution; anti‐α‐SMA from Abcam, UK, catalog ab5649 1:100 dilution; anti‐IL‐6 from Proteintech, USA, catalog 21865‐1‐AP, 1:200 dilution). Secondary antibodies were incubated under the same conditions after washing with TBST thrice (goat anti‐rabbit IgG Alexa Fluor 488, catalog ab150077, and goat anti‐mouse IgG Alexa Fluor 594, catalog ab150116; both from Abcam, UK, and diluted 1:200). Finally, sections were washed with TBST thrice, and fluorescent mounting medium (Dako, Denmark) was applied before layering a cover slip. Images were taken with the Tissue FAXS Slide Scanner (TissueGnostics, Austria).

Image analysis was performed with QuPath, using their in‐built cell detection algorithm with the following parameters: background radius = 13 µm, median radius = 0 µm, sigma = 3.0, minimum area = 3.6 µm^2^, maximum area = 722.5 µm^2^, threshold = 10, cell expansion = 8.5 µm, smooth boundaries = true. Subsequent analysis was then performed on Python (pandas v1.5.3, numpy 1.24.0, scipy v1.10.0, statsmodels v0.13.5), which was also used to plot Figure S3C,D (Supporting Information) (matplotlib v3.7.0). Cells were first filtered by nucleus size and total DAPI signal (Figure S2C, Supporting Information), then positive signals for each marker was done based on total marker expression per cell, using Otsu's thresholding from the skimage v0.19.3 package (Figure S2D, Supporting Information).

### CAF Conditioned Media and Cancer Spheroid Viability

CAFs were cultured till they attained their state (myCAF and IL‐iCAF) and treated with paclitaxel (4 µm) or DMSO as mentioned previously. Hydrogels were then washed with PBS for 5 min and RPMI with 5% FBS for 10 min. After which, cells were incubated with RPMI with 5% FBS (1 mL per 1 × 10^5^ cells) for 24 h, and the resulting media was collected as conditioned media. Conditioned media was stored at −80 °C until use.

To form cancer spheroids, 2% agarose (30 µL, RedMan, Singapore) was pipetted into each well of a 96‐well plate. Thereafter, they were sterilized under UV for 20 min and incubated with RPMI (100 µL) with 10% FBS. Cancer spheroids were then formed by adding 1.5 × 10^4^ cancer cells per well and incubating for 24 h in 100 µL of the same media. This allowed cancer cells to form spheroids of about 100 µm in diameter (Figure S2F, Supporting Information). Media was then carefully removed, and spheroids were encapsulated in Matrigel (3.5 mg mL^−1^, Corning, USA). Conditioned media from the untreated CAFs (equivalent amount of DMSO) were then applied for 48 h, then that from paclitaxel‐treated CAFs were applied for another 48 h. A control using fresh RPMI with 5% FBS was also used to normalize the data. Cancer spheroid viability was assessed using CellTiter‐Glo (Promega, USA) with a similar protocol as above, with mechanical agitation to disrupt Matrigel.

### Kaplan‐Meier Analysis

The cBioPortal website was utilized^[^ ^84^ ^]^ to download the clinical information and RNAseq data of head and neck cancer patients involved in the TCGA study.^[^ ^54^ ^]^ Only HPV^−^ patients were included in the analysis. We then identified those that had been treated with paclitaxel, docetaxel, both or neither, and stratified them according to their enrichment of CAF subset gene signatures. Patients in the bottom one‐third, middle one‐third, and top one‐third were classified as low, medium, and high, respectively. We used the survival package version 3.3‐1^[^ ^85^ ^]^ to conduct the Kaplan‐Meier analysis, and the survminer package version 0.4.9^[^ ^86^ ^]^ to generate the plots.

### Statistical Analysis

All bioinformatics analyses were performed using R version 4.2.0^[^ ^60^ ^]^ unless otherwise specified, and graphs and figures were plotted using ggplot2 version 3.4.2,^[^ ^61^ ^]^ ComplexHeatmap version 2.12.1,^[^ ^62^ ^]^ networkD3 version 0.4,^[^ ^63^ ^]^ fmsb version 0.7.5,^[^ ^64^ ^]^ circlize version 0.4.15,^[^ ^65^ ^]^ and the base R packages. Microscopy images were processed and analyzed with Fiji/ImageJ version 1.54f,^[^ ^66^ ^]^ and no complex adjustments were made except uniformly altering brightness when signals were lower. Biological experiments involved 3 patient‐derived CAF lines, and each experiment was conducted with at least 3 replicates. For statistical analyses, the Wilcoxon rank sum test was used when the experimental results contained no equal values, and the t test otherwise, unless otherwise specified. These tests were also unpaired, unless stated otherwise. When multiple tests were done, p values were adjusted to the false discovery rate. Statistical significance was defined as *p* < 0.05.

## Conflict of Interest

N.G.I. sits on the Scientific Advisory Boards of PairX Therapeutics, VerImmune and Vivo Surgical, and has received honoraria/funding from Merck, Kalbe Biotech and Agilent, all of which are outside the scope of this submitted work.

## Author Contributions

N.C.W.H., E.L.S.F., and N.G.I. conceptualized this study. N.C.W.H., J.Y.Y.Y., Z.Z., C.L., X.L.K., H.S.Q., C.A., E.L.S.F., and N.G.I. developed methodologies. Experiments were performed by N.C.W.H. (all experiments), Y.W. (confocal microscopy), and K.F. (tumor explant culture). N.C.W.H. performed formal analysis and visualization, and acquired software. E.L.S.F. and N.G.I. supervised this study. N.C.W.H. wrote the original draft of the manuscript. N.C.W.H., C.H.L., E.L.S.F., and N.G.I. wrote, reviewed, and edited the final manuscript.

## Supporting information

Supporting Information

Supplemental Table 1

Supplemental Table 2

Supplemental Table 3

Supplemental Table 4

Supplemental Table 5

Supplemental Table 6

Supplemental Table 7

## Data Availability

All code is available on github.com/nccsCancerTherapeuticsLab/HNSCC_NicHo. All raw counts data have been or will be uploaded on Gene Expression Omnibus (GEO). Raw counts data for scRNAseq can be obtained under accession number GSE188737 and from another separate repository once the paper that first used the data is published (refer to aforementioned GitHub website for updates). GEO accession number for bulk RNAseq counts will also be updated on the GitHub website upon publication.
