# Supplementary material for: Bioengineered Hydrogels Recapitulate Fibroblast Heterogeneity in Cancer
Source: Adv Sci (Weinh). 2024 Mar 17;11(20):2307129. doi: 10.1002/advs.202307129 (PMC11132030; doi:10.1002/advs.202307129)
Supplement: Supplementary file 1 — Supporting Information [file ADVS-11-2307129-s002.pdf]

ADVANCED SCIENCE
Open Access

Supporting Information

for *Adv. Sci.*, DOI 10.1002/advs.202307129

Bioengineered Hydrogels Recapitulate Fibroblast Heterogeneity in Cancer

Nicholas Ching Wei Ho, Josephine Yu Yan Yap, Zixuan Zhao, Yunyun Wang, Kanishka Fernando, Constance H Li, Xue Lin Kwang, Hong Sheng Quah, Camille Arcinas, N. Gopalakrishna Iyer and Eliza Li Shan Fong**

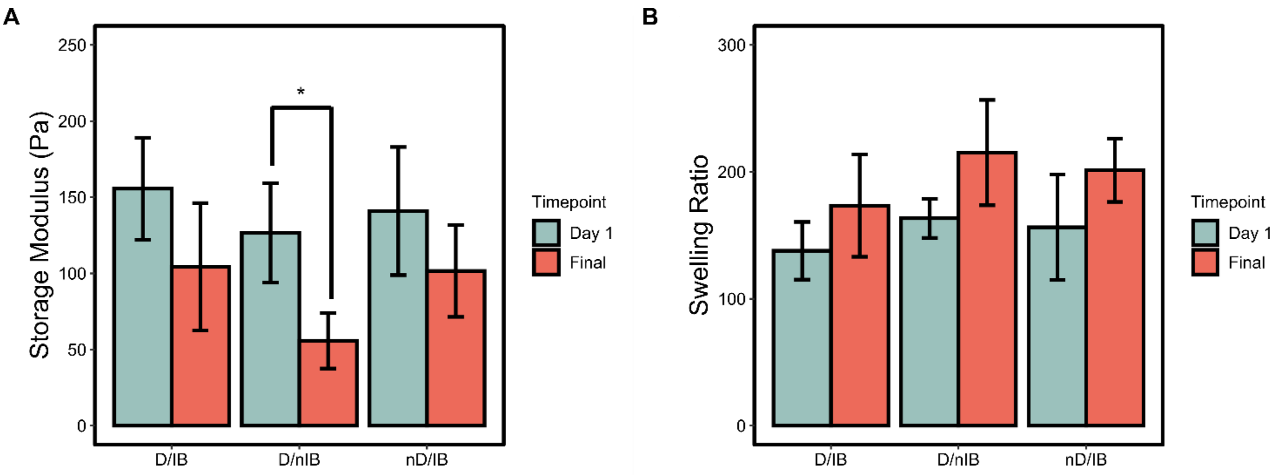

Figure S1. Biophysical characterization of hydrogels. Biophysical characterization of hydrogels (n=6; pairwise two-sided Wilcoxon rank-sum test, *$p < 0.05$). Changes in stiffness (A) and swelling ratio, which is proportional to hydrogel pore size (B). Data presented as mean ± SD.

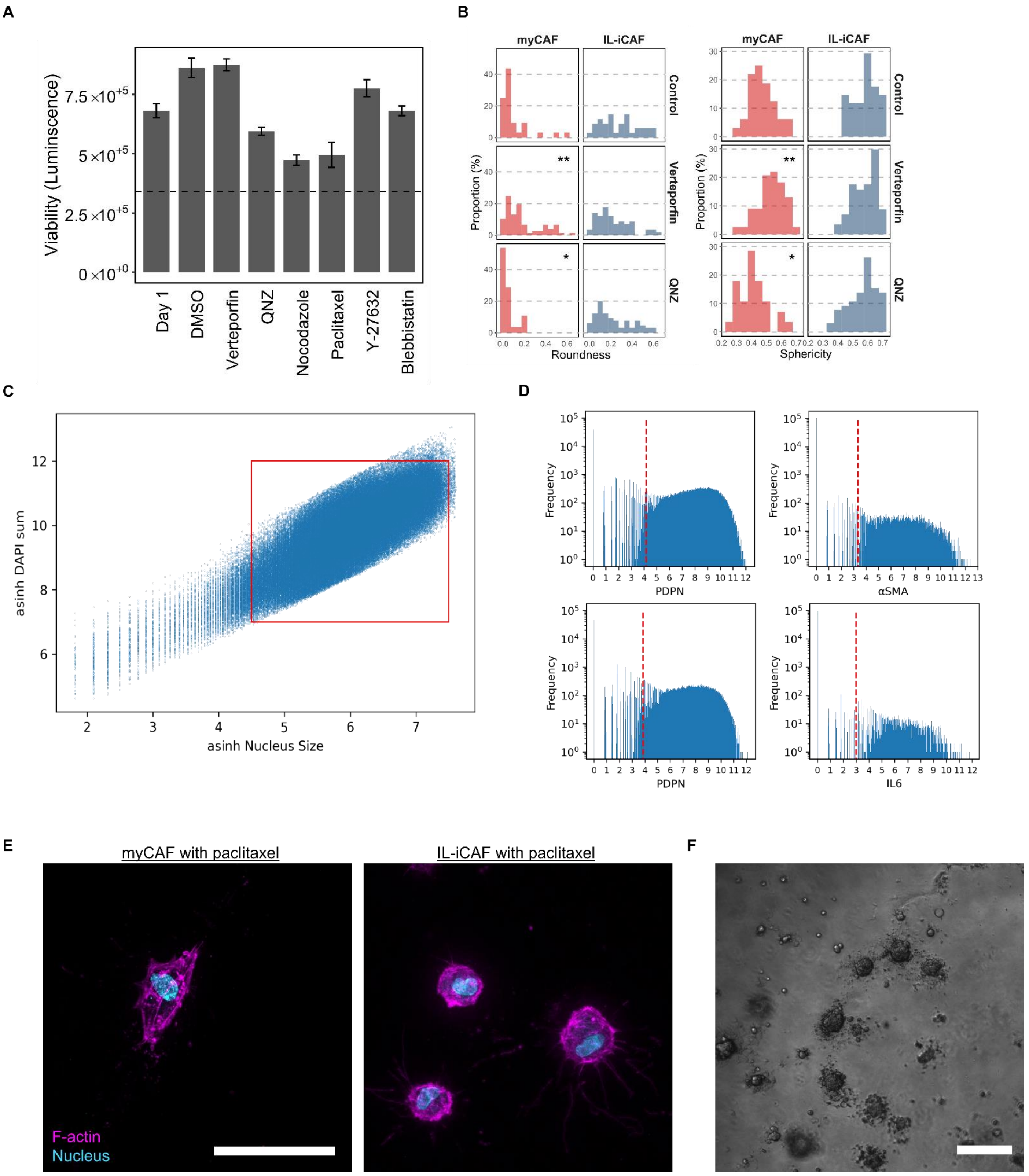

Figure S2. Further characterization of drug-treated CAFs. (A) Cell viability, in luminescence, was measured using Cell Titer-Glo®. Dashed line represents 50% of initial (Day 1) viability. Drug concentrations used were 1 µM verteporfin, 10 µM QNZ, 1 µM nocodazole, 4 µM paclitaxel, 50 µM Y-27632, and 20 µM blebbistatin. Data presented as mean ± SD (n=6). (B) Changes in morphology of verteporfin- and QNZ-treated CAFs. Pairwise, two-sided Wilcoxon rank-sum test, $^*p < 0.05$, $^{**}p<0.01$ when compared to respective controls. (C) Scatter plot of asinh normalized nuclei size and sum of DAPI signal per nucleus. Red box represents thresholds used to filter for correctly detected nuclei using the QuPath software. (D) Histograms of the total expression of PDPN, α-SMA, or IL-6 per cell. Red dashed lines represent thresholds use to classify cells as being $PDPN^+\alpha\text{-}SMA^+$ for myCAFs (top panel) and $PDPN^+IL\text{-}6^+$ for IL-iCAFs. (E) High magnification images (100x) of CAFs treated with paclitaxel, stained for F-actin and nucleus. White bar represents 50 µm. (F) Representative image of cancer spheroids used in conditioned media experiments. White bar represents 200 µm.

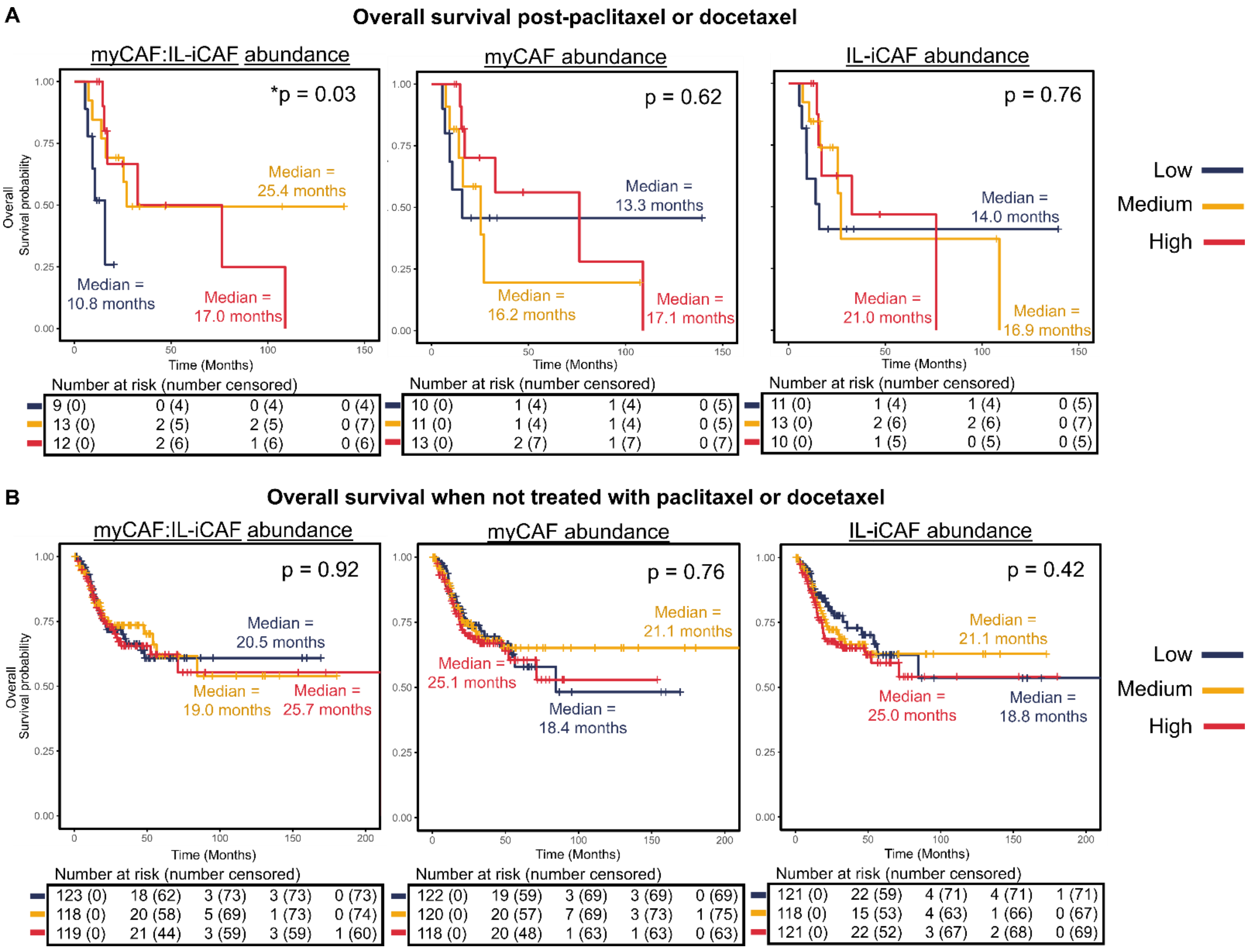

A – myCAF:IL-iCAF abundance, number at risk (number censored)

Low	9 (0)	0 (4)	0 (4)	0 (4)
Medium	13 (0)	2 (5)	2 (5)	0 (7)
High	12 (0)	2 (6)	1 (6)	0 (6)

A – myCAF abundance

Low	10 (0)	1 (4)	1 (4)	0 (5)
Medium	11 (0)	1 (4)	1 (4)	0 (5)
High	13 (0)	2 (7)	1 (7)	0 (7)

A – IL-iCAF abundance

Low	11 (0)	1 (4)	1 (4)	0 (5)
Medium	13 (0)	2 (6)	2 (6)	0 (7)
High	10 (0)	1 (5)	0 (5)	0 (5)

B – myCAF:IL-iCAF abundance

Low	123 (0)	18 (62)	3 (73)	3 (73)	0 (73)
Medium	118 (0)	20 (58)	5 (69)	1 (73)	0 (74)
High	119 (0)	21 (44)	3 (59)	3 (59)	1 (60)

B – myCAF abundance

Low	122 (0)	19 (59)	3 (69)	3 (69)	0 (69)
Medium	120 (0)	20 (57)	7 (69)	3 (73)	1 (75)
High	118 (0)	20 (48)	1 (63)	1 (63)	0 (63)

B – IL-iCAF abundance

Low	121 (0)	22 (59)	4 (71)	4 (71)	1 (71)
Medium	118 (0)	15 (53)	4 (63)	1 (66)	0 (67)
High	121 (0)	22 (52)	3 (67)	2 (68)	0 (69)

Figure S3. Effects of the interaction between CAF states and microtubule-targeting drugs on survival. (A-B) Kaplan-Meier (logrank) analysis HNSCC patients that were (A) or were not (B) treated with paclitaxel or docetaxel, then stratified by myCAF:IL-iCAF abundance (left), myCAF abundance (middle), and IL-iCAF abundance (right). *p < 0.05.
